# Statement of Retraction: Dependence on linkers’ flexibility designed for benzenesulfonamides targeting discovery of novel hCA IX inhibitors as potent anticancer agents

**DOI:** 10.1080/14756366.2025.2511543

**Published:** 2025-06-04

We, the Publisher of the *Journal of Enzyme Inhibition and Medicinal Chemistry*, have retracted the following article:

**Tawfik, H. O., Belal, A., Abourehab, M. A. S., Angeli, A., Bonardi, A., Supuran, C. T., & El-Hamamsy, M. H. (2022). Dependence on linkers’ flexibility designed for benzenesulfonamides targeting discovery of novel hCA IX inhibitors as potent anticancer agents. *Journal of Enzyme Inhibition and Medicinal Chemistry*, *37*(1), 2765–2785. https://doi.org/10.1080/14756366.2022.2130285**

Following publication, the following concerns regarding the article were brought to the Publisher’s attention by a third party:

- Apparent unexpected similarities between Figure 4 of the above article and Figure 8 of the following article from an unrelated author group:
  Alhakamy NA, Fahmy UA. Exploring cytotoxicity of cordycepin loaded nanovesicles against (HCT116) colon cancer cells: Optimization and cellular evaluation. Biomed Pharmacother. 2022 Oct;154:113619. doi: 10.1016/j.biopha.2022.113619. Epub 2022 Sep 5.
- Apparent unexpected similarities between Figure 5 of the article and Figure 2 of the following article from an unrelated author group:
  Hu Z, Fang H, Wang X, Chen D, Chen Z and Wang S: Overexpression of SHP2 tyrosine phosphatase promotes the tumorigenesis of breast carcinoma. Oncol Rep 32: 205-212, 2014.

Concerns were additionally identified regarding the integrity of the flow cytometry plots shown in Figure 6 of the article.

The Publisher queried the authors about these concerns and the authors have fully cooperated with the investigation. The authors explained that these experiments described in the article were outsourced, and were done by an external party who was paid for these services. However, this outsourced work was not declared at submission or in the published article. The authors have not been able to provide the original data.

As verifying the validity of published work is core to the integrity of the scholarly record, we are therefore retracting the article. The corresponding author listed in this publication has been informed. The authors do not agree with the retraction.

We have been informed in our decision-making by our editorial policies and the COPE guidelines.

The retracted article will remain online to maintain the scholarly record, but it will be digitally watermarked on each page as ‘Retracted’.

